# CU06-1004-Induced Vascular Normalization Improves Immunotherapy by Modulating Tumor Microenvironment *via* Cytotoxic T Cells

**DOI:** 10.3389/fimmu.2020.620166

**Published:** 2021-01-26

**Authors:** Songyi Park, Ji Hoon Oh, Dong Jin Park, Haiying Zhang, Minyoung Noh, Yeomyung Kim, Ye-Seul Kim, Hyejeong Kim, Young-Myeong Kim, Sang-Jun Ha, Young-Guen Kwon

**Affiliations:** ^1^ Department of Biochemistry, College of Life Science and Biotechnology, Yonsei University, Seoul, South Korea; ^2^ R&D Department, Curacle Co. Ltd, Seongnam-si, South Korea; ^3^ Vascular System Research Center, Kangwon National University, Chuncheon, South Korea

**Keywords:** CU06-1004, anti-PD-1, combination therapy, drug delivery, tumor microenvironment (TME), immune checkpoint blockade (ICB), vascular leakage blocker, cytotoxic T lymphocytes

## Abstract

Blocking the immune evasion mechanism of tumor cells has become an attractive means for treating cancers. However, the usage of a drug such as nivolumab (αPD-1), which blocks programmed cell death protein 1 (PD-1), turned out to be only effective against certain types of cancer. Especially, vascular abnormal structures of which deter delivery route by leakage and cause the poor perfusion were considered to be environment unfavorable to T cells and immune checkpoint blockade (ICB) delivery within the tumor microenvironment (TME). Herein, we report stabilization of tumor blood vessels by endothelial dysfunctional blocker CU06-1004, which modified the TME and showed synergistic effects with immunotherapy anti-PD-1 antibody. CU06-1004 combination therapy consistently prolonged the survival of tumor-bearing mice by decreasing tumor growth. T-cell infiltration increased in the tumors of the combination group, with cytotoxic CD8^+^ T cell activity within the tumor parenchyma upregulated compared with anti-PD-1 monotherapy. Tumor inhibition was associated with reduced hypoxia and reduced vessel density in the central region of the tumor. These effects correlated significantly with enhanced expression of IFN gamma and PD-L1 in tumors. Taken together, our findings suggest that CU06-1004 is a potential candidate drug capable of improving therapeutic efficacy of anti-PD-1 through beneficial changes in the TME.

## Introduction

Immunotherapy is an innovative way of treating cancers. However, response rates and effects vary among tumors expressing specific biomarkers (1, 2). Therefore, immunosuppression may be a critical factor contributing to tumor growth (3). Tumors can also evade T cells by expressing immunosuppressive molecules or receptors, such as programmed death-ligand 1 (PD-L1) (4). Programmed cell death 1 (PD-1) binds PD-L1 and is expressed on the surface of immune cells, including activated T cells, B cells, monocytes, natural killer (NK) cells, and dendritic cells (5). PD-1/PD-L1 interactions trigger intracellular signaling, preventing immune cell activation and cytokine secretion (6). Immune checkpoint blockade (ICB) targeting PD-L1/PD-1, galectin-9/TIM-3, IDO1, LAG-3, and CTLA4 seeks to increase the number and activity of CTLs in tumors (7). These therapies share some properties but commonly face drug delivery challenges (8, 9). Enhanced delivery technologies, increased interactions of drugs with target proteins, and reduced extratumoral drug release, may improve efficacy (10).

The solid tumor microenvironment (TME) is a major cause of tumor progression and treatment resistance (11). Oncotic pressure, acidosis, and hypoxia interfere with normal functions and promote immunological and metabolic changes (12, 13). TME changes are strongly associated with abnormalities in tumor vessels, including reduced vascular density and structurally disordered expansion (14). These changes may result in hyper-permeable vasculature (15). Endothelial cells support tumor cell apoptosis by accommodating the movement of CTLs and oxygen to the TME (16). Immunosuppression created by the abnormal TME vasculature cannot be modified by anti-PD-1 therapy (17), which may restore TME structure but does not abolish blood vessels (18). Thus, vascular stabilization by cancer treatment may increase the efficacy of immunotherapy by normalizing blood vessel perfusion (19).

CU06-1004 is a small molecule that sustains vascular stabilization and prevents endothelial loss (20–22). CU06-1004 forms cortical actin rings through cAMP/Rac/Cortactin and strengthens the endothelial barrier, inhibiting vascular leakage (20). In addition, CU06-1004 regulates various factors such as vascular endothelial growth factor (VEGF), histamine, and thrombin and decreases IL-1β cytokine level. CU06-1004 also reduces vascular hyper-permeability and tumor hypoxia (21). In an *in vivo* model of ischemic stroke, injection of CU06-1004 inhibited IL-1β-induced endothelial permeability and NF-κB activation, significantly reducing neurological deficits, cerebral infarction, and glial activation (22). Additionally, in a model of tumor angiogenesis, CU06-1004 normalized the tumor vasculature and improved delivery and efficacy of the anti-angiogenesis drug sunitinib (20). This drug combination, which directly regulates expression of vascular junction proteins, pericytes, and smooth muscle actin to induce vascular normalization, may overcome tumor progression and treatment resistance (20). Surprisingly, CU06-1004 injection improved direct drug delivery to tissue by blocking disruption of vascular structure and maximizing binding to target proteins (20–22).

Here, we examine the impact of CU06-1004, an endothelial dysfunctional blocker, on the TME in MC38 tumor models in combination with an anti-PD-1 antibody. We demonstrate that direct drug delivery of CU06-1004 results in tumor-infiltrating immune cell populations containing NK and T cells and tumor apoptosis induced by increased CD8^+^ T cell activity. Analysis of tumor cytokines showed that IFNγ expression was highly regulated within the TME. Conversely, NK and T cell depletion experiments showed that the CD8^+^ T cell population in the tumor was important for the combination of CU06-1004 and ICB therapy. Our findings indicate that CU06-1004, which blocks endothelial dysfunction, improves efficacy of ICB in the TME compared with anti-PD-1 therapy alone.

## Materials and Methods

### Mice

Male C57BL/6 mice, aged 7–8 weeks, were purchased from DBL Korea under semi-SPF conditions. All experiments were approved by the committee (IACUC-A-20201015-117).

### Drugs

Anti-PD-1 immunotherapy (*InVivo*MAb anti-mouse PD-1 (CD279); RMP1-14; BE0146) and IgG2a control antibody (*InVivo*MAb rat IgG2a isotype control; 2A3; BE0089) were purchased from BioXcell Korea. A stock solution of anti-PD-1 and isotype control (2 mg/ml) in PBS was used for dilutions. CU06-1004 was previously reported (20–22). To synthesize CU06-1004, a tetrahydropyran analog was prepared by reacting dihydropyran and pregnenolone in p-toluenesulfonic acid. After Wittig olefination using 4- (carboxybutyl) triphenylphosphonium bromide, acid-moiety methylation was performed by trimethylsilyl diazomethane. CU06-1004 was synthesized *via* tetrahydropyran deprotection with subsequent glycosidation with 4, 6-di-O-acetyl-2, 3-didieoxyhex-2-enopyran in the presence of acid (23).

### Cell Culture

MC38 colon adenocarcinoma cells (kind gift from Prof. Sang-Jun Ha; Yonsei University, Seoul, Korea) were cultured in complete Dulbecco’s modified Eagle’s media (DMEM; Hyclone; SH30022.01) supplemented with 10% fetal bovine serum (FBS; GE Healthcare UK Ltd) and 1% penicillin/streptomycin (Gibco Laboratories) at 37°C in a 5% CO_2_ incubator in a humidified atmosphere.

### *In Vivo* Models

Tumors were subcutaneously implanted into the right flanks of 7- to 8-week-old C57BL/6 mice. Tumor volumes were measured every 2 or 3 days according to the formula (0.523 x (length x width^2^)). The drug was injected approximately 1 week after the tumor was implanted.

### Anti-PD-1 and CU06-1004 Treatment

*InVivo*Mab rat IgG2a isotype control antibody 200 μg/dose was injected intraperitoneally (i.p.) in vehicle and CU06-1004-alone groups on days 0, 3, and 6 after all tumors were visible. Similarly, *InVivo*Mab anti-mouse PD-1 antibody 200 μg/dose was injected i.p. in the anti-PD-1-alone and combination anti-PD-1 plus CU06-1004 groups on days 0, 3, and 6 after all tumors were visible. CU06-1004 1mpk (1 mg/kg) was injected intravenously (i.v.) in the CU06-1004-alone and combination group during daily a week at the same time point as the anti-PD-1 drug.

### Immunofluorescence Staining

Mice were anesthetized with i.p. 2.5% avertin and then perfused with 50 ml PBS or saline *via* the left ventricle of the heart. Whole tumors were collected, fixed with 4% paraformaldehyde (PFA) for 16 hours (h), dehydrated in 15% sucrose solution, and followed by a 30% sucrose solution until tumors sank to the bottom of the container. Mouse tumor tissues were sectioned 20–30 μm thick using a cryostat (Leica, Wetzlar, Germany). One of every 7 to 10 slices was collected. Sections were stored at -80°C. To examine vascular leakage, tumor tissue slices were permeabilized in 0.5% PBS Triton X-100 (PBST) for 5 minutes (min), incubated in blocking solution at room temperature (RT) for 1 h, and incubated with primary antibodies for double staining of CD31/PECAM1 (R&D systems; AF3628; 1:200, Santa Cruz, SC-1506; 1:200) with VE-cadherin (Santa Cruz; SC-9989; 1:200), α-SMA (Abcam; ab7817; 1:200), NG2 (Millipore; 92950; 1:200), or collagen IV (Millipore; 1982483; 1:200) at 4°C for 16 h and then at RT for 1 h. To examine increased T cells in the tumor, double staining of CD31/PECAM1 with CD8 (Abcam; ab22378; 1:200) or CD3 (Abcam; ab16669; 1:200) was performed at 4°C for 16 h and then at RT for 1 h. After washing, slides were incubated with the appropriate Alexa-Flour 488-, 594-conjugated secondary antibodies (1:500) at RT for 1 h. For nuclear staining, slides were treated with DAPI (1:1000) for 20 min before mounting. Immunofluorescence was imaged using confocal microscopy (Carl Zeiss 700, Germany). Quantification of fluorescence intensity and cell counting were performed using Image J (NIH) or Photoshop version CS6 (Adobe Systems, San Jose, CA). Data represent twice independent experiments.

### Phycoerythrin-Anti-PD-1 Staining

CU06-1004 was injected daily i.v. for a week in the CU06-1004-treated group,** **and PE anti-mouse CD279 (PD-1) (BioLegend; RMP1-14; 114118) 100 μg/dose was injected i.v. in vehicle and CU06-1004-alone groups. Mice were harvested and perfused on day 7. All tumors were fixed in 4% PFA and dehydrated in 15% sucrose solution, 30% sucrose solution, and followed by sectioning (20–30 μm thick). Nuclei in tumor tissue slices were stained with DAPI (1:1000) for 20 min and mounted. Immunofluorescence was imaged using confocal microscopy (Carl Zeiss 700, Germany). Quantification of fluorescence intensity and cell counting were performed using Image J (NIH).

### *In Situ* Apoptosis Detection

Fresh tumor tissue was immediately frozen in optimal cutting temperature (OCT) compound and sectioned onto a *silanized slide*. Tumor tissue slices were washed with PBS for 20 to 30 min. Labeling reaction mixture plus 100 μl permeabilization buffer was maintained on ice for 2 to 5 min. The labeling reaction mixture (50 μl) consisting of 5 μl TdT enzyme and 45 μl labeling-safe buffer was incubated on the slide at 37°C for 60 to 90 min. To prevent drying, the glass slide was covered with a plastic coverslip. The reaction was terminated, and the slide was washed 3 times in PBS for 5 min each time. The tumor tissue slide was analyzed by confocal microscopy (Carl Zeiss 700, Germany).

### Cell Preparation

Cells from spleen and peripheral blood were isolated as described (24). Spleens were passed through a 70-µm cell strainer (BD Falcon), and red blood cells were lysed using ACK lysing buffer (Gibco Laboratories). Peripheral blood from the retro-orbital sinus was underlaid with Histopaque-1077 (Sigma-Aldrich). The gradient was centrifuged at 2000 rpm without braking for 20 min at 20°C, and mononuclear cells were recovered from the interface. To isolate tumor-infiltrating lymphocytes, tumors were removed, weighed, and chopped into small pieces. Following incubation in 1 mg/ml collagenase IV (Worthington Biochemical Corp.) and 0.01 mg/ml DNase (Sigma-Aldrich) in RPMI containing 10% FBS and 1% penicillin/streptomycin at 37°C for 30 min, digested cells were passed through a 70-µm cell strainer (BD Falcon), and red blood cells were lysed using ACK lysing buffer (Gibco Laboratories). After washing with RPMI containing 2% FBS and 1% penicillin/streptomycin, cells were counted using a hemocytometer.

### Flow Cytometry and Antibodies

For flow cytometry analysis, cells in single-cell suspension were plated in each well of 96-well plate(round-bottom) which also contains PBS consisting of 2% FBS. The cells were stained with fluorochrome-conjugated antibodies for 20 min in 4°C. Antibodies used for cell labelling were BV605 anti-CD45.2 (BioLegend Cat. No. 109841, clone: 104), BV421 anti-CD4 (BioLegend Cat. No. 100544, clone: RM4-5), BV421 anti-CD11c (BioLegend Cat. No. 117329, clone: N418), APC anti-PD-L1 (BioLegend Cat. No. 124312, clone: 10F.9G2), PerCP-Cy5.5 anti-CD107a (BioLegend Cat. No. 121626, clone: 1D4B), PE-Cy7 anti-CD25 (eBioscience Cat. No. 25-0251-82, clone: PC61.5), PE-Cy7 anti-CD8a (eBioscience Cat. No. 25-0081-82, clone: 53-6.7), PerCP-Cy5.5 anti-CD8a (eBioscience Cat. No. 45-0081-82, clone: 53-6.7), FITC anti-F4/80 (eBioscience Cat. No. 11-4801-82, clone: BM8), PE anti-Foxp3 (eBioscience Cat. No. 12-4771-80, clone: NRRF-30), PE-Cy7 anti-CD11b (BD Biosciences Cat. No. 552850, clone: M1/70), APC anti-NK1.1 (BD Biosciences Cat. No. 554420, clone: PK136), PerCP-Cy5.5 anti-Ly6G (BD Biosciences Cat. No. 565797, clone: 1A8), Alexa Fluor 488 anti-Ki-67 (BD Biosciences Cat. No. 561165, clone: B56), PE anti-NK1.1 (BD Biosciences Cat. No. 553165, clone: PK136) FITC anti-IFNγ (BD Biosciences Cat. No. 554411, clone: XMG1.2), and APC anti-TNF (BD Biosciences Cat. No. 554420, clone: MP6-XT22). The LIVE/DEAD fixable dead cell stain kit (Invitrogen) was used to remove the dead cell population in all staining procedures. Additionally, for intracellular staining process, cells were first stained with surface antigens followed by permeabilization and fixation with either Foxp3 Fixation/Permeabilization Kit and Protocol (eBioscience) or Cytofix/Cytoperm Kit (BD Biosciences) according to the manufacturer’s instructions before the appropriate antibodies were added. All the stained samples were analyzed using FACSCantoII instrument (BD Biosciences) and FlowJo software (Tree Star).

### Cytokine Analysis

To detect cytokines, spleen and tumor cells were either stimulated with MC38 epitope peptide (p15E, KSPWFTTL) or with 25 ng/ml phorbol myristate acetate (PMA) and 1 μM ionomycin (Sigma-Aldrich) in the presence of Golgi plug/Golgi stop (BD Biosciences) and CD107a (BioLegend; clone: 1D4B) antibody at 37°C for 5 h. Subsequently, surface staining was performed, and cells were permeabilized with Cytofix/Cytoperm Kit (BD Biosciences) solution to stain intracellular cytokines.

### Reverse Transcriptase-Polymerase Chain Reaction and Quantitative PCR

Tumor tissue was harvested in 1 ml Trizol and stored at −80°C until processing. Total RNA was isolated from tumor tissues, and cDNA was synthesized using Moloney murine leukemia virus reverse transcriptase. RT-PCR was performed with a cDNA template, primer, dNTP, 10χ buffer, and Taq polymerase. And qRT-PCR was performed with SYBR Green (Invitrogen) in a Bio-Rad real-time PCR detection system. Data represent three independent experiments. Primers were: *Ifnγ*, 5’-GCTTTGCAGCTCTTCCTCAT-3’, 5’-GTCACCATCCTTTTGCCAGT-3; *Tnfα*, 5’-CCAGACCCTCACACTCACAA-3’, 5’-GTGGGTGAGGACACGTAGT-3; *Il-1β*, 5’-GGGCCTCAAAGGAAAGAATC-3’, 5’-TACCAGTTGGGGAACTCTGC-3’; *Ifnα&β*, 5’-ATGGGCAGTGTGACCTTTTC-3’, 5’-CCCTTCCTCTGCTCTGACAC-3’; *Tgfβ*, 5’-TGCGCTTGCAGAGATTAAAA-3’, 5’-CTGCCGTACAACTCCAGTGA-3’; *Gapdh*, 5’-ACCCAGAAGACTGTG GATGG-3’, 5’-CACATTGGGGGTAGGAACAC-3’.

### Western Blotting

Tumor tissues were washed with cold PBS, harvested in cytosolic buffer (10 mM Tris [pH 7.5], 0.05% NP-40, 3 mM MgCl2, 100 mM NaCl, 1 mM EGTA, 1 mM Na3VO4), and centrifuged at 15,000 rpm for 15 min. After centrifugation, nuclei were pelleted and suspended in nuclear buffer (1 mM EDTA, 3.5% SDS, 10% glycerol, and 70 mM Tris-Cl) as previously described. Proteins were separated by SDS polyacrylamide gel electrophoresis. Immunoblotting was performed with antibodies to VE-cadherin, *α*-SMA, pSTAT1, STAT1, PD-L1, and β-actin (Santa Cruz Biotechnology, Santa Cruz, CA). Data represent three independent experiments.

### *In Vivo* Depletion of Immune Cells

To deplete NK, CD4+, or CD8+ T cells, 200 μg *InVivo*MAb anti-mouse NK1.1 (BioXCell; PK136; BE0036), 200 μg *InVivo*MAb anti-mouse CD4 (BioXCell; GK1.5; BE0003-1) or 200 μg *InVivo*MAb anti-mouse CD8a (BioXCell; 2.43; BE0061) was injected i.v. twice for 2 weeks before tumor injection and before drug injection. Depletion was evaluated by flow cytometry 7 days after drug treatment. Data represent twice independent experiments.

### Live Imaging of Lewis Lung Cancer -Green Fluorescent Protein Tumor-Bearing Mice

A dorsal skinfold chamber model was constructed by injecting 1 χ 10^6^ LLC-GFP mouse lung carcinoma cells into BALB/c-nu/nu mice. Diameter and density were measured daily in groups injected with drugs and vehicle 7 days after tumor injection, and permeability was measured daily 10 days after tumor injection. Anti-CD31 antibody conjugated to a fluorescent dye was injected i.v. Vessel diameter and density were measured before drug and vehicle treatment and after 4, 24 h, 3, and 7 days. Permeability was measured 4 days after drug or vehicle injection.

### *In Vitro* Cell Cytotoxicity

Cell viability and proliferation were compared by 3- (4,5-dimethylthiazol-2-yl) -2,5-diphenyl tetrazolium-bromide (MTT) assay. MC38 cells were seeded in 24-well plates (2 χ 10^4^ cells/well). After treatment with CU06-1004 and anti-PD-1 or isotype control, cells were maintained for 48 h in media containing 0.2% FBS. MTT (0.5 mg/ml) was added to each well, and cells were incubated at 37°C for 3 h. The supernatant was removed, and 200 μl DMSO + isopropyl alcohol was added to dissolve the formazan product. Absorbance, which is proportional to the number of living cells and proliferation rate, was measured at 540 nm on a microplate reader (FLUOstar Omega, BMG LABTECH). Data represent four independent experiments.

### Enzyme-Linked Immunosorbent Assay

To analyze cytokine levels between drug-treated groups, protein was collected from each tumor tissue. Quantification was performed with BCA protein reagent (SMART™ BCA Protein Assay Kit Solution A and B; iNtRON BIOTECHNOLOGY; 21071) and RIPA buffer assay (cOmplete ULTRA Tablets; Roche). IFNγ (Mouse IFN-gamma DuoSet ELISA; R&D Systems; DY485) and TNF*α* (Mouse TNF-alpha DuoSet ELISA; R&D Systems; DY410) were measured by ELISA kit. Data represent three independent experiments.

### Survival Analysis

Standard Kaplan–Meier survival analysis was used to determine associations with survival, with 1 indicating alive and 0 dead. Data represent three independent experiments.

### Statistical Analysis

Data are presented as mean ± standard error of the mean (SEM). All statistical analyses were performed using GraphPad Prism (version 8; GraphPad Software, La Jolla, CA). The mean difference between groups was analyzed by two-way ANOVA to reveal differences in tumor growth rates. The mean difference between groups was also analyzed by one-way ANOVA. *p < 0.05; **p < 0.01; ***p < 0.001; ****p < 0.0001. ns, not significant.

## Results

### CU06-1004 Aids Anti-PD-1-Mediated Tumor Growth Inhibition and Overall Survival

**Figure 1:**
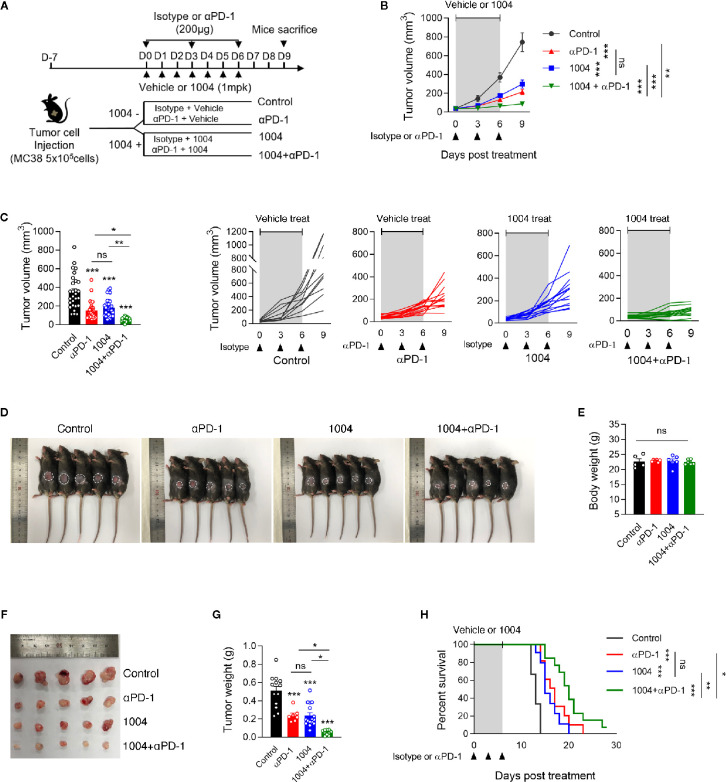
Combined Vascular leakage blocker, CU06-1004 and anti-PD-1 delayed MC38 tumor growth and extended mice survival. MC38 tumor cells (5 × 10^5^ cells/mouse) were injected subcutaneously into the right flank of C57BL/6 mice. At 7 days post tumor inoculation (tumor size <100 mm^3^), tumor-bearing mice were treated with control IgG, anti-PD-1, CU06-1004, or anti-PD-1 + CU06-1004. The control IgG or anti-PD-1 was treated 200ug intraperitoneally once every 3 days (total 3 times), and CU06-1004 was treated intravenously at 1 mg/kg daily. **(A)** Schematic diagram depicting treated schedule for the MC38 tumor-bearing mice model. **(B, C)** Tumor size was measured every 3 days from the start of the treatment. Growth curve graphs show mean (top) and individual (bottom) tumor growth and bar graphs show tumor volume on 6-days post treatment. B, *n* = 10–14 per group and C, *n* = 23–28 per group (Data are pooled from three B, or six C, independent experiments). Statistical analysis by two-way B, or one-way C, ANOVA with Tukey’s multiple comparisons. **(D, E)** At 7 days post treatment, Body weight were measured from each group of mice. Scale bars indicate 1 cm. *n* = 5-6 per group (Data are representative of at least four independent experiments). Statistical analysis by one-way ANOVA with Tukey’s multiple comparisons. **(F, G)** At 7 days post treatment, tumor weight were measured from each group of mice. Scale bars indicate 1 cm. *n* = 7–16 per group. Statistical analysis by one-way ANOVA with Tukey’s multiple comparisons. **(H)** Kaplan-Meier survival curves of MC38 tumor-bearing mice treated as indicated. Mice were euthanized when the mean tumor size reached 2000mm^3^. *n* = 9-13 per group. *p < 0.05; **p < 0.01; ***p < 0.001. ns, not significant. Data represent ± SEM.

To observe the synergistic effect of using immune checkpoint blockade drug and CU06-1004, which is a drug known for its high ability to normalize blood vessel, we conducted monotherapy or combinatory therapy to MC38 tumor-bearing mice with anti-PD-1 and CU06-1004. Four different treatment groups were decided depending on types of drug administered (**Figure 1A**). As treatment began, tumor volumes were measured, and some mice were sacrificed seven days after the day of initial treatment to further measure the tumor weight difference. The growth of tumor volume between these groups of mice showed that the combination therapy effectively controlled the tumor progression compared to the other three groups (**Figures 1B–D**). Since the tumor volume of mice in combination group were maintained in small volume, the size and the weight were the lowest as well (**Figures 1E, F**). As it can be expected from the increment of tumor volume and weight, the mouse that were treated with both CU06-1004 and anti-PD-1 showed higher survival rate than any other mouse groups (**Figure 1G**). The suppression of tumor growth using combination therapy was also observed when the drugs were administered for longer period of time (**Figure S1A**). In the case of anti-PD-1 monotherapy, the rate of tumor growth increased significantly after 12 days, which indicated loss of drug efficiency. However, in combination group it inhibited the tumor progression considerably (**Figure S1B**). Also looking at the survival outcome, while anti-PD-1 monotherapy did not show any differences between short- and long-term treatment, group that received combination therapy showed noticeable survival benefit even in long-term treatment period (**Figure S1C**). In addition to MC38 tumor model, CT26 tumor model was also used to demonstrate the effect of CU06-1004 in other tumor model that do not respond to anti-PD-1(**Figure S2**). This became the basis for showing the importance of blocking blood vessel leakage and vascular normalization in the early stages in changing the tumor microenvironment.

### CU06-1004 Obstructs Vascular Collapse by Maintaining Blood Vessel Structure and Prevents Uncontrolled Angiogenesis Within Central Tumor Region in MC38 Tumors

**Figure 2:**
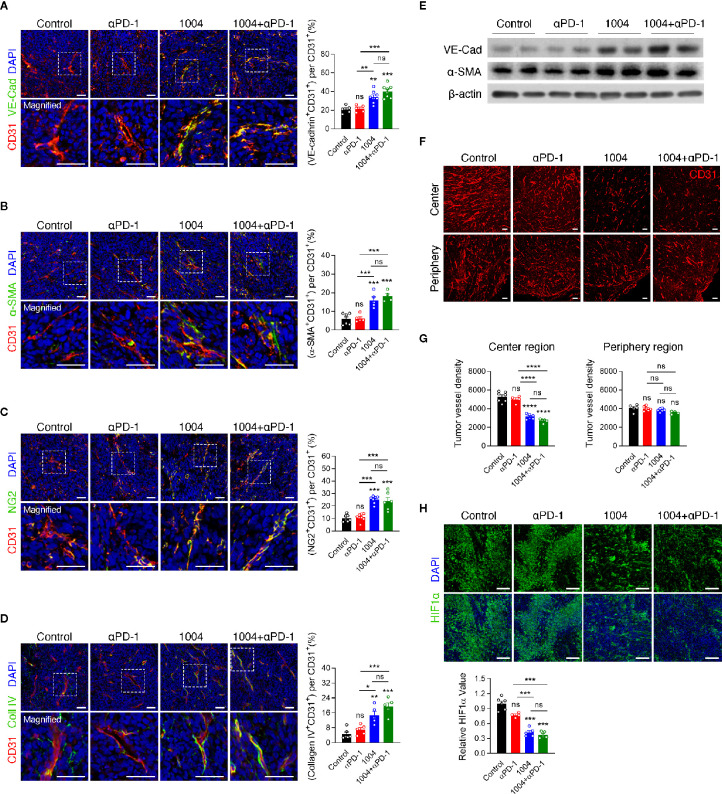
CU06-1004 improved tumor vascular normalization and decreased hypoxia and abnormal vessel density. **(A)** Representative immunofluorescence staining for adherent junction coverage (the ratio VE-cadherin^+^CD31^+^/CD31^+^ area) on day 7. Red, CD31 staining; green, VE-cadherin staining; blue, DAPI staining, Scale bars; 20 µm. *n* = 5-8 per group. Statistical analysis by one-way ANOVA with Tukey’s multiple comparisons. **(B)** Representative immunofluorescence staining for α-smooth muscle actin coverage (the ratio αSMA^+^CD31^+^/CD31^+^ area) on day 7. Red, CD31 staining; green, αSMA staining; blue, DAPI staining, Scale bars; 20 µm. *n* = 4-6 per group. Statistical analysis by one-way ANOVA with Tukey’s multiple comparisons. **(C)** Representative immunofluorescence staining for pericyte coverage (the ratio NG2^+^CD31^+^/CD31^+^ area) on day 7. Red, CD31 staining; green, NG2 staining; blue, DAPI staining, Scale bars; 20 µm. *n* = 6-8 per group. Statistical analysis by one-way ANOVA with Tukey’s multiple comparisons. **(D)** Representative immunofluorescence staining for collagen IV (the ratio Collagen IV^+^ CD31 ^+^/CD31^+^ area) on day 7. Red, CD31 staining; green, Collagen IV staining; blue, DAPI staining, Scale bars; 20 µm. *n* = 5-6 per group. Statistical analysis by one-way ANOVA with Tukey’s multiple comparisons. **(E)** Western blotting for adherent junction protein of VE-cadherin and αSMA protein in tumor tissue on day 7. **(F)** The entire blood vessel density was stained in the tumor center region and periphery region. Red, CD31 staining, Scale bars; 100 µm. **(G)** Quantification of the vessel density in the MC38 tumor center region and periphery region. *n* = 4–7 per group. Statistical analysis by one-way ANOVA with Tukey’s multiple comparisons. **(H)** Representative images of HIF1α^+^ area in core tumor region on day 7. Green, HIF1α staining; blue, DAPI staining, Scale bars; 100 µm. Quantification of the hypoxic area on day 7. *n* = 4-6 per group. Statistical analysis by one-way ANOVA with Tukey’s multiple comparisons. *p < 0.05; **p < 0.01; ***p < 0.001; ****p < 0.0001. ns, not significant. Data represent ± SEM.

Tumor regression observed in combination group showed that CU06-1004 can complement the effect of anti-PD-1 treatment significantly. Thus, these results prompted us to see if blood vessel normalization effect of CU06-1004 can also be observed in MC38 tumor model which may be the reason for improved immunotherapy response. We previously reported that CU06-1004 inhibits vascular leakage in endothelial cells and maintains stabilization of human umbilical vein endothelial cells (20). The effect of CU06-1004 was measured using immunofluorescence staining and first, the markers for vessel and adherent junctions were selected which included CD31/PECAM1, and VE-Cadherin, respectively. CD31 and VE-Cadherin were clearly detected and seem to form a normal vessel structure only when CU06-1004 is given (**Figure 2A**). Additionally, other markers that are related to vessel structures such as α-SMA, NG2, and collagen IV were measured. The fluorescent figures indicate that intact vessel structures are present only in groups that are treated with CU06-1004 (**Figures 2B–D**). To further confirm these fluorescent data, western blot was conducted and showed significant increase in VE-Cadherin and a-SMA (**Figure 2E**). Lastly, by injecting Evans blue into the tumor tissue of CU06-1004 untreated and treated group, 20-s time-lapse movie was made. The perfusion of Evans blue into the vessels showed that in CU06-1004 treated group, the chemical is dispersed following the blood vessel without any leakage (**Figure S3** and **Figure SV1**). These results showed that vascular leakage was prevented by inhibiting structural deformation. Treating CU06-1004 not only kept the vessels intact but also seem to have prevented abnormal and uncontrolled angiogenesis. Vessel density within the tumor central region was significantly decreased when CU06-1004 was treated. In contrast, the blood vessel density in peripheral region did not show any difference (**Figures 2F, G**). Since the abrupt angiogenesis and vessel breakage were prevented, we postulated that transportation of substances such as oxygen would be more effective. In fact, assessment of HIF1α protein marker which indicates hypoxia level showed that the level of hypoxia decreased in CU06-1004 treated group (**Figure 2H**).

### Effective Trafficking of Anti-PD-1 Drug Through Normalized Blood Vessels Expands Tumor Infiltrated Lymphocytes

**Figure 3:**
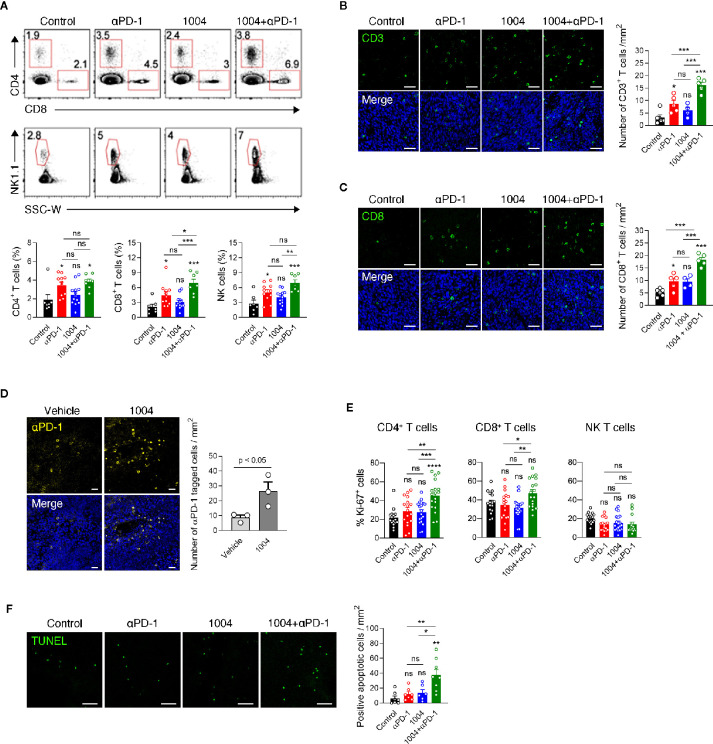
Combined CU06-1004 and anti-PD-1 treatment promotes the accumulation of specific CD8^+^ T cells and apoptosis in tumors. **(A)** Flow cytometry analysis of the CD4^+^(CD45.2^+^CD3^+^NK1.1-CD8-CD4^+^), CD8+ (CD45.2^+^CD3^+^NK1.1-CD8^+^CD4-), T and NK cells (CD45.2^+^CD3-NK1.1^+^), in subcutaneous MC38 tumors from each group of mice on 7 days post treatment. Numbers in the representative flow cytometric plots indicate the percentages of each cell type among CD45.2+ cells. Data are pooled from two independent experiments (Control, *n* = 7; αPD-1, *n* = 11; CU06-1004, *n* = 13; Combination, *n* = 7). Statistical analysis by one-way ANOVA with Tukey’s multiple comparisons. **(B)** Representative immunofluorescence staining for CD3^+^T cells infiltration in the center region on day 7. Green, CD3 staining; blue, DAPI staining, Scale bars; 50 µm. Quantification of infiltrated T cells on day 7. *n* = 4–6 per group. Statistical analysis by one-way ANOVA with Tukey’s multiple comparisons. **(C)** Representative immunofluorescence staining for CD8^+^ T cells infiltrating MC38 tumor on day 7. Red, CD31 staining; green, CD8 staining; blue, DAPI staining, Scale bars; 50 µm. Quantification of infiltrated CD8^+^ T cell numbers in MC38 tumor on day 7. *n* = 4–6 per group. Statistical analysis by one-way ANOVA with Tukey’s multiple comparisons. **(D)** Representative immunofluorescence staining for PE-conjugated anti-PD-1 in the tumor on day 7. Yellow, PE-anti-PD-1; blue, DAPI staining, Scale bars; 20 µm. PE-conjugated anti-PD-1 intravenously treated one day before harvesting a tumor. *n* = 3 per group. Statistical analysis by one-way ANOVA with Tukey’s multiple comparisons. **(E)** Representative graph showing the percentages of Ki-67^+^ cells among CD4^+^, CD8^+^ T and NK cells. Data are pooled from five independent experiments (Control, *n* = 16; αPD-1, *n* = 17; CU06-1004, *n* = 19; Combination, *n* = 18). Statistical analysis by one-way ANOVA with Tukey’s multiple comparisons. **(F)** Representative immunofluorescence staining for apoptotic areas in the tumor on day 7. Green, TUNEL staining, Scale bars; 50 µm. *n* = 6–8 per group. Statistical analysis by one-way ANOVA with Tukey’s multiple comparisons. *p < 0.05; **p < 0.01; ***p < 0.001. ns, not significant. Data represent ± SEM.

Next, in order to see whether tumor vessel normalization induced by CU06-1004 enhanced the infiltration of lymphocyte from periphery into tumor central region, we conducted flow cytometry analysis of tumor infiltrating lymphocyte (TIL) retrieved from mice sacrificed at 7 days after the treatment. Compared to the control group, the percentage of CD4, CD8 and NK cells was significantly increased in the anti-PD-1 monotherapy group and combination treatment group, but not in the CU06-1004 monotherapy group. Additionally, the comparison between anti-PD-1 monotherapy group and combination therapy group showed increased frequency of CD8 T cell in combination treatment group, excluding CD4 and NK cells (**Figure 3A**). Immunofluorescence staining data also supported the flow cytometry data by showing accumulation of CD3^+^ and CD8^+^ T cell in tumor site in anti-PD-1 monotherapy group and combination treatment group, but not in the monotherapy group. (**Figures 3B, C**). The frequency of T cell in the tumor center region with many abnormal blood vessels showed a significant difference by combination treatment, but the frequency of T cell in the relatively stable structure of the tumor periphery region did not show a significant difference between groups (**Figure 3B** and **Figure S4**). In contrast to the effective transportation of oxygen to the tumor site through normalized blood vessels (**Figure 2**), the accumulation of T cells in tumor site between the CU06-1004 monotherapy group and the control group was slight difference, indicating that CU06-1004 treatment alone does not lead to significant infiltration of immune cells into tumor site. Thus, we hypothesized that treatment of CU06-1004 allows the trafficking of anti-PD-1 into tumor central region *via* normalized vessels which leads to the expansion of tumor infiltrated lymphocytes instead of increasing the ability of effective infiltration of immune cells. To assess the trafficking efficacy of anti-PD-1 drug *via* normalized blood vessel, immunofluorescence staining was conducted with tumor tissue that was injected with PE-tagged anti-PD-1 antibody at the 6 days instead of anti-PD-1 treatment. Based on the data, more anti-PD-1 drug was detected in CU06-1004 treated group than that of control group and anti-PD-1 antibody implied that vessel normalization by CU06-1004 treatment induces increased trafficking of drug to the lymphocyte within the tumor site. (**Figure 3D**). Since anti-PD-1 drug has been known to rejuvenate and induce expansion of lymphocyte within tumor, the proliferation of T cells was assessed by analyzing Ki67 expression level. As expected, the increased Ki67 expression level of T cells in combination treatment group was observed which indicated proliferation of T cells by anti-PD-1 drug (**Figure 3E**). Additionally, TUNEL assay was conducted and the results indicated that the number of apoptotic cells were increased in combination therapy group which suggests that increment of cytolytic immune cells could lead to induction of apoptosis in cancer cells (**Figure 3F**). Additionally, neither CU06-1004 nor anti-PD-1 alone affected tumor cell viability (**Figure S5**).

### Combination Therapy Enhances the Function of Tumor Infiltration CD8^+^ T Cells

**Figure 4:**
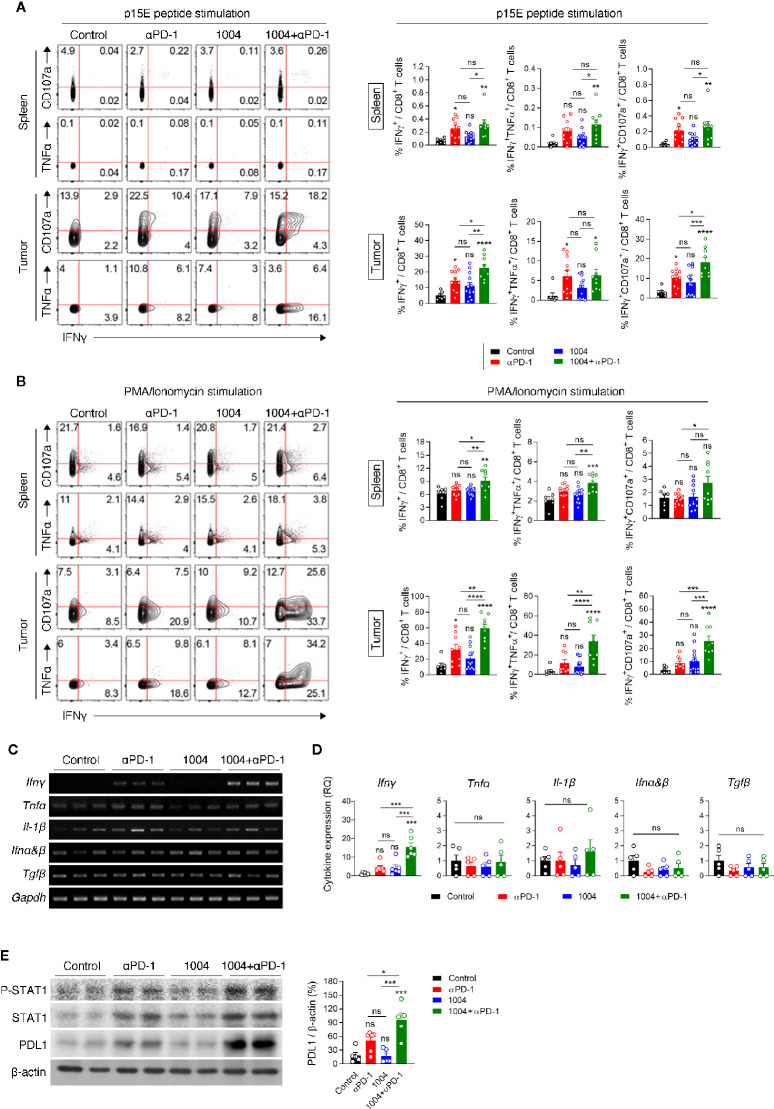
Combination therapy improves tumor specific CD8^+^ T cell response in spleen and tumor tissue. **(A, B)** Lymphocytes isolated from the spleen and tumor of each group of mice at 7 days post treatment were re-stimulated *in vitro* with MC38 epitope peptide **(A)** or PMA/Ionomycin **(B)**. Representative plots (left) are shown for co-expression of IFNγ and CD107a or IFNγ and TNFα on CD8^+^ T cells; summarized graph (right) for the frequency of IFNγ^+^, IFNγ^+^TNFα^+^ and IFNγ^+^CD107a^+^ cells among CD8^+^ T cells. Data are pooled from three independent experiments (Control, *n* = 7; αPD-1, *n* = 9; CU06-1004, *n* = 13; Combination, *n* = 9). Statistical analysis by one-way ANOVA with Tukey’s multiple comparisons. **(C)** RT-PCR analysis for cytokine and chemokine expression in tissue after drug-treated tumor harvest. **(D)** qRT-PCR analysis of inflammatory cytokines *Ifnγ, Tnfα, Il-1β, Ifnα&β*, and *Tgfβ* in tumor tissue. *n* = 5–6 per group. Statistical analysis by one-way ANOVA with Tukey’s multiple comparisons. **(E)** Western blotting analysis of p-STAT1, STAT1, and PD-L1 expression in tumor tissue. *n* = 5-6 per group. Statistical analysis by one-way ANOVA with Tukey’s multiple comparisons. *p < 0.05; **p < 0.01; ***p < 0.001; ****p < 0.0001. ns, not significant. Data represent ± SEM.

Increased frequency of CD8^+^ T cells within the tumor of combination therapy group led us to analyze the functionality of TIL by measuring intracellular cytokines and cytotoxic marker CD107a. Lymphocytes from spleen and tumor were re-stimulated with either p15E peptide, which is a known CD8^+^ T cell-specific antigen of MC38 tumor, or with PMA and Ionomycin. Although the expression level was not significant between anti-PD-1 and combination group, stimulating these two groups of spleen cells with p15E showed statistically significant IFNγ expression compared to control group. In contrast to the splenocytes, when tumor infiltrated lymphocytes (TIL) are stimulated with p15E, much higher percentage of IFNγ and CD107a expressing cells were observed. Also, TILs retrieved from combination therapy group showed the highest frequency of IFNγ^+^ and IFNγ^+^CD107a^+^CD8^+^ T cells compared to other treatment groups (**Figure 4A**). These results indicate that combination therapy allows tumor antigen-specific T cells to be maintained in the tumor site while keeping their cytolytic activity and restrains from entering exhausted state. Stimulating the same immune cells with PMA and Ionomycin showed similar trend compared to p15E peptide stimulation. In splenic T cells, although every group expressed quite amount of IFNγ as PMA/Ionomycin was treated, the combination treatment group showed evidently higher percentage of expression. IFNγ, TNFα and CD107a expression levels in TILs were dramatically increased compared to expression levels in splenic T cells. In addition, CD8^+^ T cells in combination treatment group not only higher expression level of IFNγ but also showed higher frequency of CD8^+^ T cells expressing TNFα or CD107a with higher frequency than CD8^+^ T cells retrieved from other treatment groups. (**Figure 4B**). In sharp contrast to the effect of combination therapy, monotherapy of either drug had not shown such expression level of cytokines from CD8^+^ T cells. In addition, expression of IFNγ was also measured in CD4^+^ T cells and in NK cells in the spleen and tumor. CD4^+^ T cells in the spleen and tumor did not show a significant difference in IFNγ expression between each group. Similarly, there was no difference in the expression level of IFNγ in NK cells from the spleen, but NK cells retrieved from tumors treated with combination therapy showed some different expression level (**Figure S7A**). These results indicates that many functional CD8^+^ T cells are present in tumor due to combination therapy of CU06-1004 and anti-PD-1. We also observed high expression level of IFNγ in tumor from combination therapy group by performing RT-PCR, QPCR and ELISA (**Figures 4C, D** and **Figure S6**). Looking at IFNγ down signaling markers by western blot showed that p-STAT1, STAT1 and PD-L1 were prominently higher in combination group where expression level of IFNγ were very high (**Figure 4E** and **Figure S8**). These results indicate that the secretion of IFNγ by T cells in the tumor from combination group is physiologically increased.

### CD8^+^ T Cells Are Directly Involved in Controlling Progression Tumor Growth

**Figure 5:**
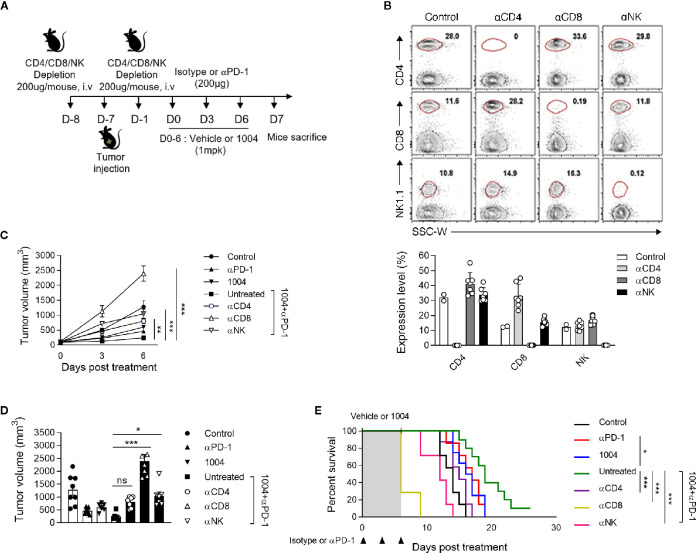
Specific CD8^+^ T cells are indispensable for suppressing tumor growth by combination therapy. **(A)** αCD4, αCD8 or αNK1.1-depleting mAb was administrated intravenously into each group of mice prior to tumor injection (0.2 mg) and prior to treatment (0.2 mg). **(B)** At day 7 after first depletion, CD4^+^, CD8^+^ T cells, and NK cells were analyzed in the PBMC from each group of mice. Representative flow cytometric plots and graphs show the frequencies of CD4^+^, CD8^+^ T cells, and NK cells in PBMC. **(C, D)** Tumor size was measured every 3 days from the start of the treatment. Growth curve graphs show mean tumor growth and bar graphs show tumor volume on 6-days post treatment. *n* = 5–6 per group. Statistical analysis by two-way **(C)** or one-way **(D)** ANOVA with Tukey’s multiple comparisons. **(E)** Kaplan-Meier survival curves of MC38 tumor-bearing mice treated as indicated. Mice were euthanized when the mean tumor size reached 2000mm^3^. *n* = 7–10 per group. *p < 0.05; **p < 0.01; ***p < 0.001. ns, not significant. Data represent ± SEM.

Since we have seen that combination therapy led to the expansion of functional CD8^+^ T cells in the tumor, we sought to determine if tumor suppression is indeed dependent on CD8^+^ T cells by depleting each immune cell type including CD4^+^ T, CD8^+^ T cells, and NK cells. The experiment was conducted using same scheme as previous experiments except depletion antibody was injected twice, once a day before tumor injection and the other on the day before drug injection (**Figure 5A**). After seven days of first depletion antibody injection, we confirmed depletion efficacy of corresponding cell types in PBMC (**Figure 5B**). In combination treatment group, the depletion of any immune cell subtypes led to more rapid tumor growth compared to the non-depleted group, of which the most accelerated tumor growth rate was observed when the CD8+ T cells were depleted (**Figures 5C, D**). Such increment of tumor volume inevitably induced very poor survival rate for the group that was depleted of CD8^+^ T cells (**Figure 5E**). Additionally, we performed QPCR to determine how changed expression of IFNγ in the combined group was changed after depleting CD4+ T cells, CD8+ T cells, and NK cells. Interestingly, the results showed that the expression of IFNγ, which was increased in the combination group, was significantly reduced in the group depleted of CD8+ T cells, there was no difference in the group depleted of CD4+ T cells, and slightly different in the group depleted of NK cells (**Figure S7B**). Comprehensively, these data illustrate that tumor regression observed when treating both CU06-1004 and anti-PD-1 are caused by CD8^+^ T cells which were actively participating in controlling the tumor growth.

## Discussion

**Figure 6:**
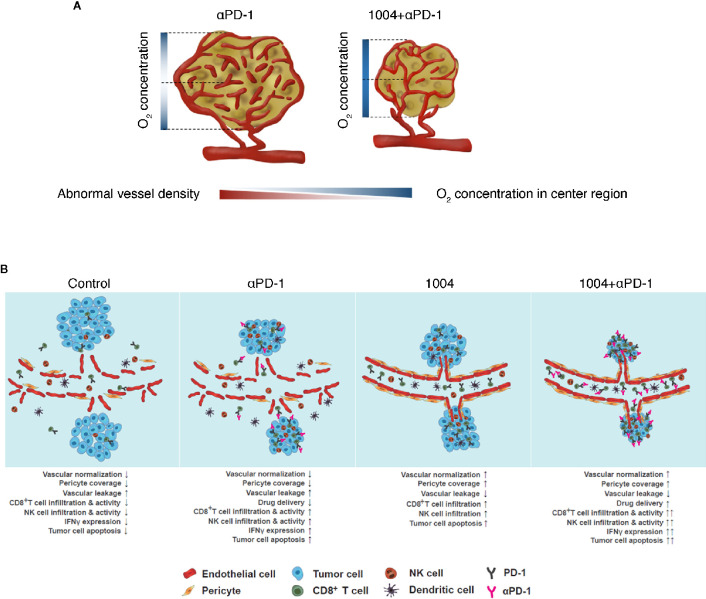
CU06-1004 enhanced the therapeutic efficacy of anti-PD-1 antibody in MC38 tumors. **(A)** In the absence of CU06-1004, the center region in the tumor had a lower O_2_ concentration and induced abnormal vascular density than the periphery region. However, when CU06-1004 was injected into the tumor, it maintained the proper O_2_ concentration in the center region as well as the periphery region, and changed it into a stable center region by reducing the relatively abnormal vascular density. **(B)** In the TME without CU06-1004 treatment, migration of immune cells was suppressed by blood vascular leakage in the tumor. When CU06-1004 was injected into the tumor, the blood vessel was normalized in tumors, decreasing vascular leakage. Penetration of immune cells was successfully achieved by the CU06-1004 injection. Production of pro-inflammatory cytokine *Ifnγ *was enhanced by CD8^+^ T cell populations after CU06-1004 injection. In conclusion, the combination of CU06-1004 and anti-PD-1 increased therapeutic efficacy in MC38 tumors.

Cancer immunotherapies that enhance T cells using blocking antibodies are more effective than chemo- or radiotherapy due to low toxicity and higher response rates (2, 25, 26). However, tumor heterogeneity, including PD-L1 expression, anti-PD-1 resistance, and TME, causes variability in treatment response (27). Leaky and hyper-permeable tumor vessels are inefficient in anti-PD-1 antibody delivery, resulting in low infiltration and activation of T cells at the tumor site (28). Despite attempts to overcome immuno-suppression by the TME, the problems remain unresolved (3, 29). In this study, CU06-1004, which normalizes the TME vasculature, was combined with ant-PD-1 immune checkpoint blockade therapy. The modified TME induced T-cell recruitment and activation, which increased the anti-tumor response. This effect was mediated by IFNγ signaling directly induced by cytotoxic CD8^+^ T cells. These effects may improve current drawbacks of PD-1/PD-L1 treatment by enhancing drug delivery and T-cell infiltration and function. Thus, combination therapy consisting of anti-PD-1 plus CU06-1004 has potential value compared with existing combination therapy (**Figure 6B**).

TME contributes to the abnormal proliferation, angiogenesis, and secretion of chemicals that promote drug resistance and induce vascular leakage and inhibition of drug delivery (15, 30, 31). Tumor vessels are irregular and highly permeable, resulting in intratumoral protein loss and fluid increase (19). Uncontrolled angiogenesis due to vessel breakage creates a favorable environment for tumor progression, including increased hypoxia (13, 32–34). However, when vascular leakage was blocked with CU06-1004 in the B16F10 melanoma mouse model, vessels were normalized, reducing hypoxia (20). Combination treatment with other anti-tumor drugs, such as doxorubicin and sunitinib, reduced tumor progression (20). Based on this observation, we used the MC38 tumor model, which is respond to anti-PD-1 therapy, and the CT26 tumor model, which is not respond to anti-PD-1 therapy and examined combination therapy using anti-PD-1 and CU06-1004. In groups treated with CU06-1004, the number of aggressive blood vessels located in the central region of the tumor mass was greatly reduced. Structural differences between the central and peripheral regions of solid tumors were significant. Vessels in the central region of solid tumors are generally unstable compared with peripheral vessels (35), reducing oxygen levels and drug transport in the central region. However, CU06-1004 reduced non-functional vessels and reduced hypoxia within the central region of the tumor mass (**Figure 6A**). These effects were observed in the MC38 cancer model and in BALB/c-nu/nu mice injected with LLC-GFP. CU06-1004 induced an anti-tumor effect similar to anti-PD-1 monotherapy. CU06-1004 did not directly affect MC38 tumor growth but caused changes in the TME that increased the number of activated cytotoxic T cells. Immune activity in tumor tissue was related to the TME changes, with hypoxia playing an important role (36). TME has a wide variety of structures depending on the type of tumor, and these TMEs are closely related to tumor vessel normalization (23). Perhaps, in ICB treatment, the difference in anti-cancer efficacy according to the type of tumor is related to differences in vascular structure (37).

Changes in T-cell function are related to increased intratumoral delivery of anti-PD-1 therapy (34). Here, we have shown that the number of cells bound to anti-PD-1 drug was lower in groups that were not treated with CU06-1004. By contrast, CU06-1004 treated groups increased the number of anti-PD-1-bound cells. CU06-1004 normalizes the vessels within the tumor mass and allows seamless movement for both the drug and immune cells. We examined changes in the T-cell population within the tumor of groups treated with both anti-PD-1 and CU06-1004. Anti-PD-1 alone can prevent PD-1/PD-L1 signaling, but only allows a portion of activated T cells to infiltrate (36). When CU06-1004 is added with anti-PD-1, the number of T cells within the tumor significantly increased due to changes in the TME. Although changing TME can influence the amount of infiltrated T cells within the tumor, it is important to assess that increased T cell population is indeed activated CD8^+^ T cells. In particular, among many T cells, CD8^+^ Tcells have been reported to directly kill tumor cells by inducing perforin/granzyme, FasL/Fas binding, or secreting cytokine/chemokine (23). Our results predict that activated CD8^+^ T cells are responsible for significant tumor suppressive effects. In order to prove that infiltrated T cells are cytotoxic CD8^+^ T cells, we analyzed FACS and fluorescence data. CU06-1004 plus anti-PD-1 increased activated T cells more than any other treatment method while unaffecting the number of regulatory T cells. Thus, combination treatment with CU06-1004 and anti-PD-1 increased the number of activated CD8^+^ T cells and improved the efficacy of immune therapy.

Generally, activated CD8^+^ T cells inhibit tumor progression by increasing the secretion of cytokines and chemokines. Cytokines secreted from CD8^+^ T cells include IFNγ, TNFα, and IL-2 (38, 39). We examined if combination treatment induced activated CD8+ T cells to express pro-inflammatory cytokines, such as IFNγ, TNFα, and IL-1β. We also checked other cytokines, such as anti-inflammatory markers IFNα, IFNβ, and TGFβ, using RT-PCR, qPCR, and ELISA. As already documented, treatment using anti-PD-1 alone can reactivate CD8+Tcells and induce cytokine secretion of tumor-antigen specific T cells. In contrast, experiments using only CU06-1004 caused lower levels of cytokine secretion compared to the anti-PD-1 treated group. Lower secretion of cytokines in the CU06-1004-treated group may be due to less activation of CD8^+^ T cells, in contrast to anti-PD-1, with only movement and infiltration enhanced. Indeed, expression of cytokines was higher in the combination group compared with the anti-PD-1 mono-treatment group, thus enhancing the anti-tumor activity of CD8^+^ T cells by combination treatment.

Defining mechanisms that affect immune response in cancer patients is important (40). Anti-PD-1 therapy strengthens T cell-derived immune responses by binding IFNγ receptors (IFNGR) in tumors, increasing PD-L1 expression through JAK1 and JAK2 activation and mobilizing surrounding STAT1 (41–43). Combination therapy compared with anti-PD-1 monotherapy showed higher expression levels of PD-L1 on cancer cells due to increased secretion of IFNγ, which increased recruitment of STAT1. Although the increase in PD-L1 on cancer cells can increase immune evasion, anti-PD-1 treatment prevented immune evasion by cancer cells and allowed T cells to mediate anti-tumor responses in our studies. A modified TME allows drugs and activated T cells to move efficiently into the central tumor region. Anti-tumor activity by these cells was supported by IFNγ expression and signaling.

Combination therapy with an immune checkpoint blocker and anti-VEGF drug inhibits angiogenesis (44, 45). Although the effect of anti-VEGF treatment may seem similar compared with CU06-1004 in that it stabilized the tumor vessels in early stage, anti-VEGF can induce hypoxia by eliminating vessels inside the tumor. By contrast, CU06-1004 normalizes vessels to yield long-lasting effects by raising oxygen and T-cell infiltration. Anti-VEGF treatment may occasionally normalize vessels, but the steady increase in hypoxia promotes tumor progression (46–48). Therefore, anti-VEGF may be effective only during early stages when a tumor is small. Combination treatment with CU06-1004 showed favorable responses at early time points of tumor development and maintained effects for a longer period. Thus, the novel combination therapy examined in this study may be effective for cancer patients, including those who require long-term treatment.

We showed that combination treatment including CU06-1004 can normalize vessels and directly change the TME, enhancing drug transfer and T-cell activity. Further, changes in IFNγ and PD-L1 expression indicate that the combination therapy is highly effective in generating an anti-tumor response. Finally, these data suggest that the combination of CU06-1004 and anti-PD-1 is highly synergistic and long-lasting with potential benefits for early and late stages of cancer.

## Conclusions

We demonstrate that the antitumor effects of CU06-1004 combined ICB through enhanced specific CD8^+^T cell activity and increased antitumor cytokine, Ifnγ. CU06-1004 used for the treatment of cancer by changing the TME and had a new promising candidate drug efficacy.

## Data Availability Statement

The original contributions presented in the study are included in the article/**Supplementary Material**; further inquiries can be directed to the corresponding authors.

## Ethics Statement

The animal study was reviewed and approved by IACUC-A-201901-843-02.

## Author Contributions

Y-GK and S-JH provided expertise and research financing, and contributed to the writing of the manuscript. SP performed all the experiments and quantifications and wrote the manuscript. JHO and DJP contributed to the experiment, quantification, and revised the manuscript. HZ, MN, YK, Y-SK, and HK helped with the experiments, and Y-MK commented. All authors contributed to the article and approved the submitted version.

## Funding

This work was supported by the Basic Science Research Program through the National Research Foundation of Korea funded by the Ministry of Education, Science and Technology (grant number 2019R1A2C3007142) and (grant number 2018R1A2A1A05076997), and the Bio & Medical Technology Development Program of the National Research Foundation of Korea (NRF-2015M3A9B6066835). The work was supported in part by Brain Korea 21(BK21) FOUR program / SP are fellowship awardee by BK21 FOUR program.

## Conflict of Interest

HZ was employed by the company Curacle Co. Ltd.

The remaining authors declare that the research was conducted in the absence of any commercial or financial relationships that could be construed as a potential conflict of interest.

## Abbreviations

1004, CU06-1004; αPD-1, anti-PD-1; TME, tumor microenvironment; IFNγ, interferon gamma; TNFα, tumor necrosis factor-alpha; IL-1β, interleukin-1 beta; IFNα& β, interferon alpha & beta; IL-10, interleukin-10; GAPDH, glyceraldehyde 3-phosphate dehydrogenase; VE-cad, vascular endothelial cadherin; α-SMA, alpha-smooth muscle actin; NG2, neuron-glial antigen 2; Coll IV, collagen IV; HIF1α, hypoxia-inducible factor 1-alpha; PMA, phorbol myristate acetate; STAT1, signal transducer and activator of transcription1; TUNEL, terminal deoxynucleotidyl transferase dUTP nick end labeling.
