# Supplementary material for: CU06-1004-Induced Vascular Normalization Improves Immunotherapy by Modulating Tumor Microenvironment via Cytotoxic T Cells
Source: Front Immunol. 2021 Jan 26;11:620166. doi: 10.3389/fimmu.2020.620166 (PMC7874050; doi:10.3389/fimmu.2020.620166)
Supplement: Supplementary file 1 [file DataSheet_1.docx]

**Supplementary Material:**

**
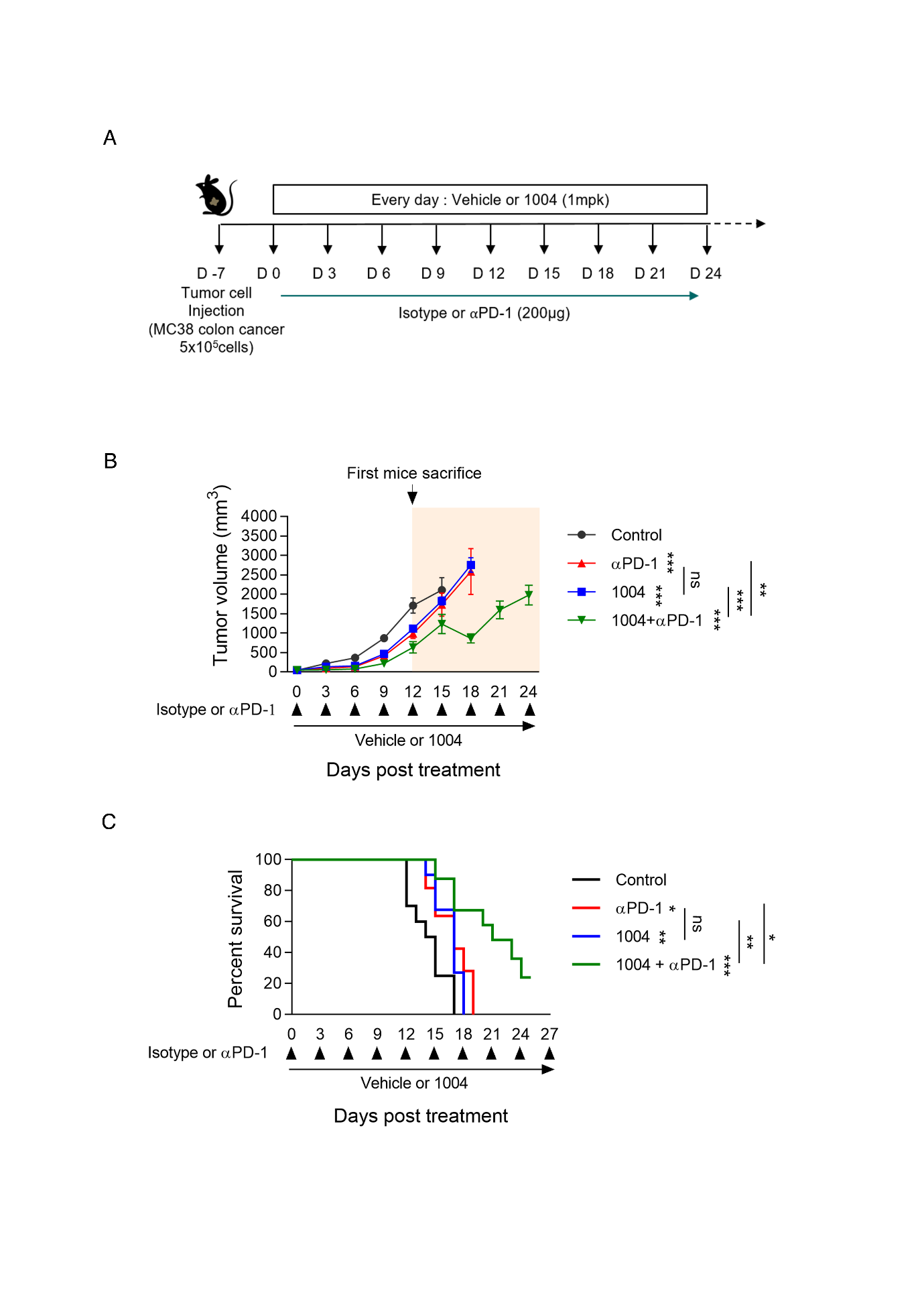
**

**Figure S1. Continuous treatment of CU06-1004 with anti-PD-1 extended the mice survival ratio.**

**(A)** Schematic of the course schedule, in which CU06-1004 and anti-PD-1 were administered daily after tumor cell injection until the tumor was 2000 mm^3^.

**(B)** Growth curves for MC38-bearing C57BL/6 mouse groups. *n* = 8-10 mice per group. Statistical analysis by two-way ANOVA.

**(C)** Survival curve for mice with MC38 tumors treated daily with 1 mg/kg control or CU06-1004 intravenously and injected intraperitoneally with 200 µg isotype control or anti-PD-1 once every 3 days. *n* = 8-10 mice per group. Statistical analysis by Kaplan–Meier.

**p* < 0.05; ***p* < 0.01; ****p* < 0.001. ns, not significant. Data represent ± SEM.


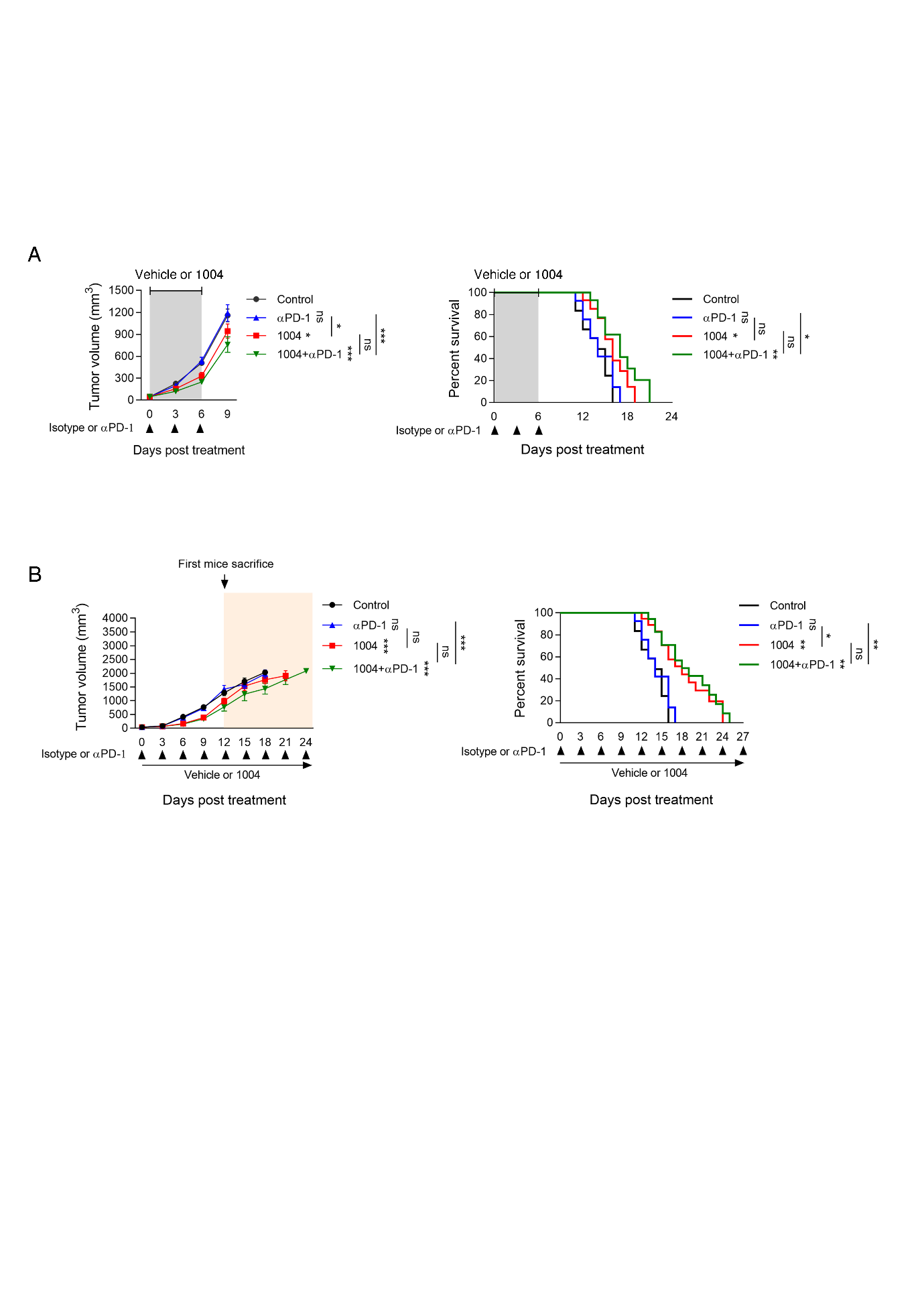


**Figure S2. The tendency of CU06-1004 is similar in other CT26 tumor models that do not respond to Anti-PD-1.**

**(A)** Tumor size was measured every 3 days from the start of the treatment. Growth curve graphs show mean tumor growth of four groups on 6 days post treatment. *n* = 10 per group. Statistical analysis by two-way ANOVA with Tukey’s multiple comparisons (Left). And kaplan-Meier survival curves of MC38 tumor-bearing mice treated as indicated. Mice were euthanized when the mean tumor size reached 2000mm^3^. *n* = 10 per group (Right).

**(B)** CU06-1004 were daily administered and anti-PD-1 were administered once every 3 days after tumor cell injection until the tumor was 2000 mm^3^. Growth curves for MC38-bearing C57BL/6 mouse groups. *n* = 10-11 mice per group. Statistical analysis by two-way ANOVA (Left). Survival curve for mice with MC38 tumors treated with 1 mg/kg control or CU06-1004 intravenously and injected intraperitoneally with 200 µg isotype control or anti-PD-1. *n* = 10-14 mice per group. Statistical analysis by Kaplan–Meier (Right).

**p* < 0.05; ***p* < 0.01; ****p* < 0.001. ns, not significant. Data represent ± SEM.

**
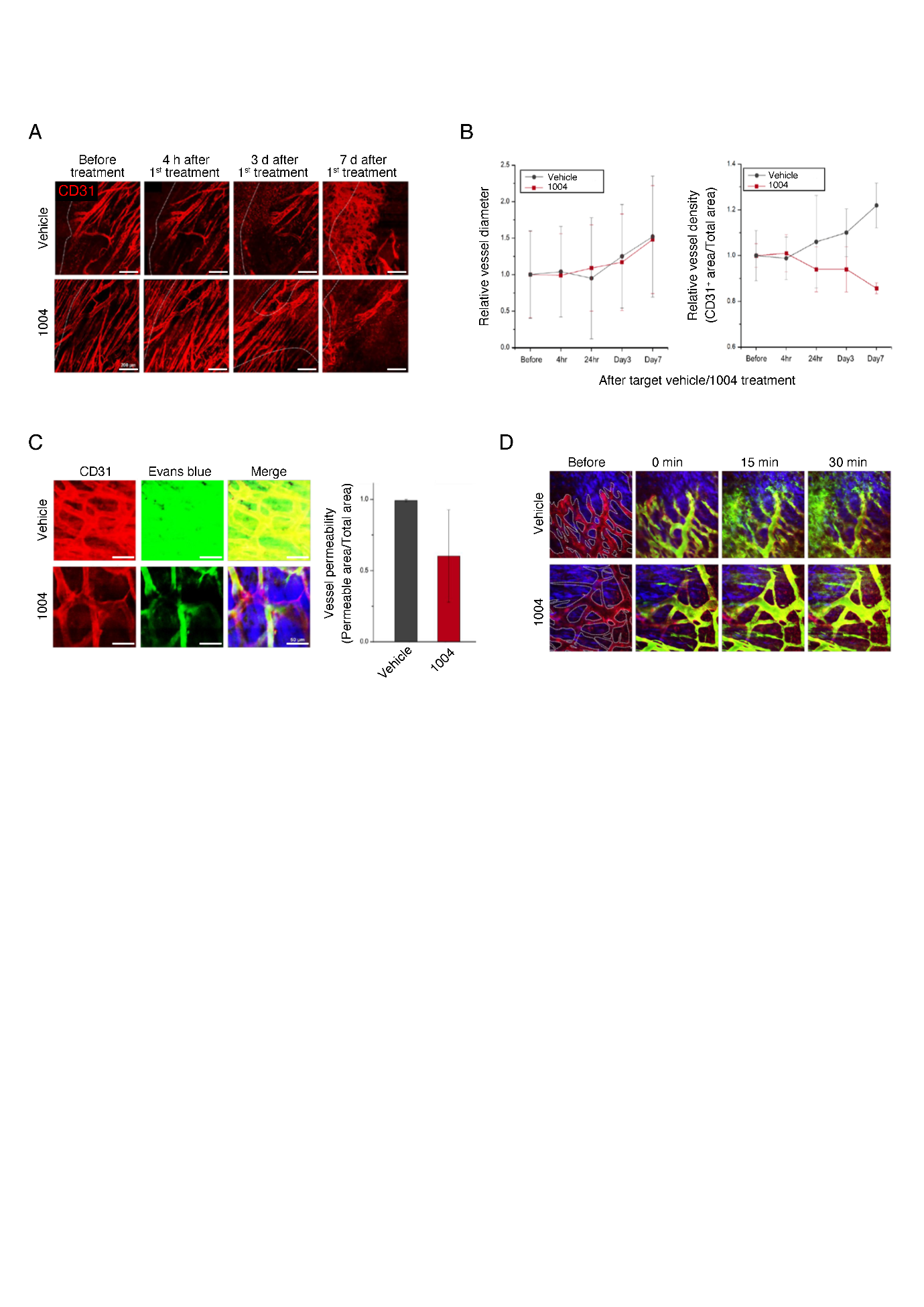
**

**Figure S3. Vascular morphology and permeability changes directly induced by CU06-1004 in the MC38 model as well as the Lewis lung cancer (LLC)-GFP model.**

**(A)** Vascular morphology changes following before vehicle or CU06-1004 treatment and 4hours, 3days, 7days after first vehicle or CU06-1004 treatment were imaged in the LLC**-**GFP model, Scale bars; 200 µm.

**(B)** Quantification of relative vessel diameter and vessel density (CD31^+^ area/ Total area) following before vehicle or CU06-1004 treatment and 4hours, 3days, 7days after first vehicle or CU06-1004 treatment were graphed in the LLC**-**GFP model.

**(C)** Vascular permeability difference (Permeable area/ Total area) by representative CD31 and Evans blue staining was imaged and quantified in LLC by the vehicle or CU06-1004 injection, Scale bars; 50 µm.

**(D)** The changes of vascular leakage following before vehicle or CU06-1004 treatment and 0min, 15min, 30min after vehicle or CU06-1004 treatment were imaged in the LLC**-**GFP model.


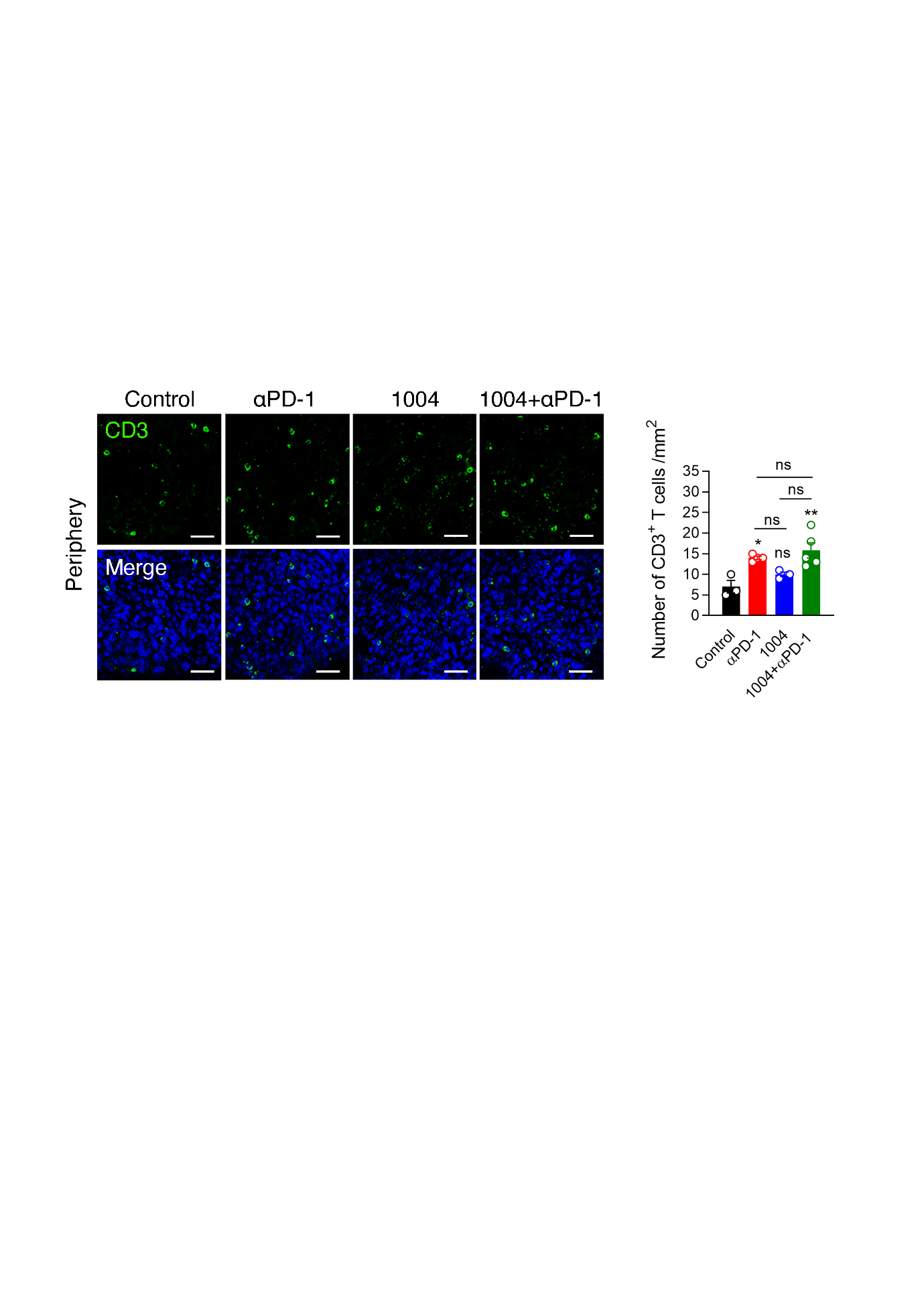


**Figure S4. T cell infiltration in a relatively stable vascular structure of periphery region does not show a significant difference between groups.**

Representative immunofluorescence staining for CD3^+^T cells infiltration in the periphery region on day 7. Green, CD3 staining; blue, DAPI staining, Scale bars; 50 µm. Quantification of infiltrated T cells on day 7. Data represent ± SEM. *, p<0.05, n=4-6 mice per group. Statistical analysis by one-way ANOVA with Tukey’s multiple comparisons.

**p* < 0.05; ***p* < 0.01; ****p* < 0.001. ns, not significant. Data represent ± SEM.


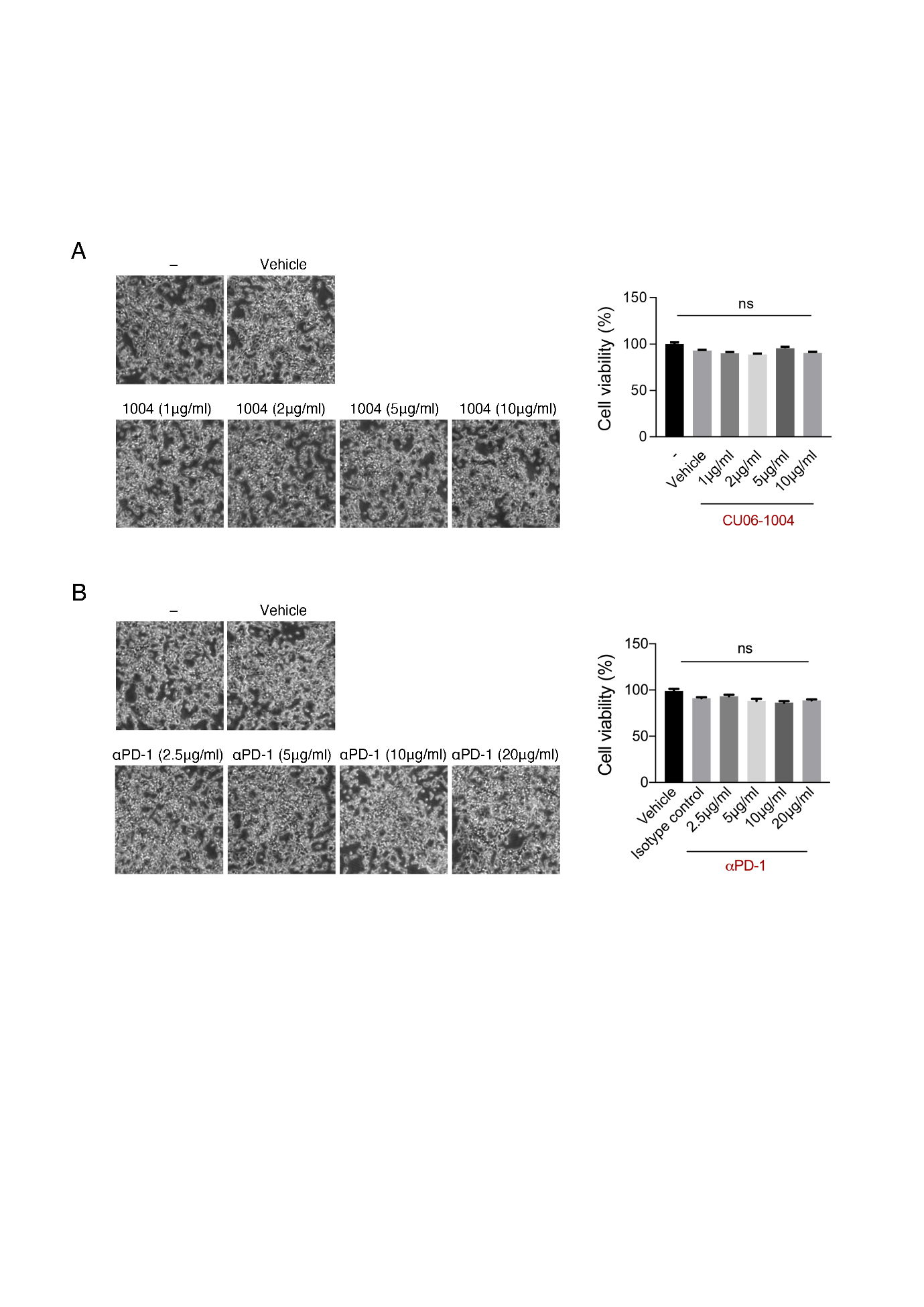


**Figure S5. CU06-1004 and anti-PD-1 by themselves did not induce drug toxicity in MC38 cancer cells in vitro.**

**(A)** CU06-1004 was injected at different concentrations but did not affect cancer. Data represent four independent experiments. Statistical analysis by one-way ANOVA with Tukey’s multiple comparisons.

**(B)** Anti-PD-1 was injected at different concentrations, but the drug alone did not show any differences. Data represent four independent experiments. Statistical analysis by one-way ANOVA with Tukey’s multiple comparisons.

**p* < 0.05; ***p* < 0.01; ****p* < 0.001. ns, not significant. Data represent ± SEM.

**
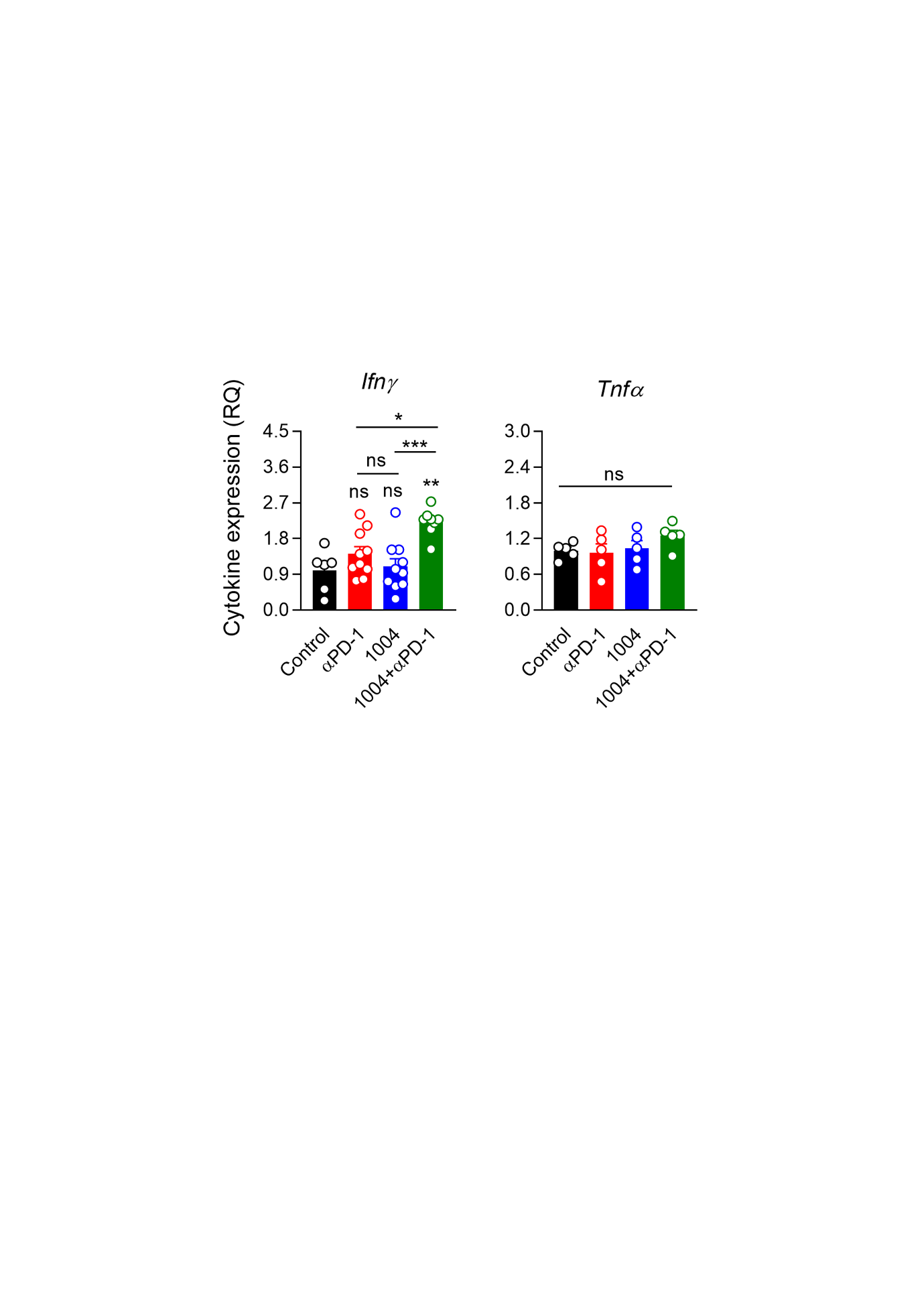
**

**Figure S6. IFNγ and TNFα expression differences in the proteins of each group were compared by ELISA.**

ELISA level of proteins by MC38 tumor precipitation demonstrated a significant difference in IFNγ, TNFα levels between the 4 groups. *n* = 6-10, *n* = 5 per group. Statistical analysis by one-way ANOVA with Tukey’s multiple comparisons.

**p* < 0.05; ***p* < 0.01; ****p* < 0.001. ns, not significant. Data represent ± SEM.

**
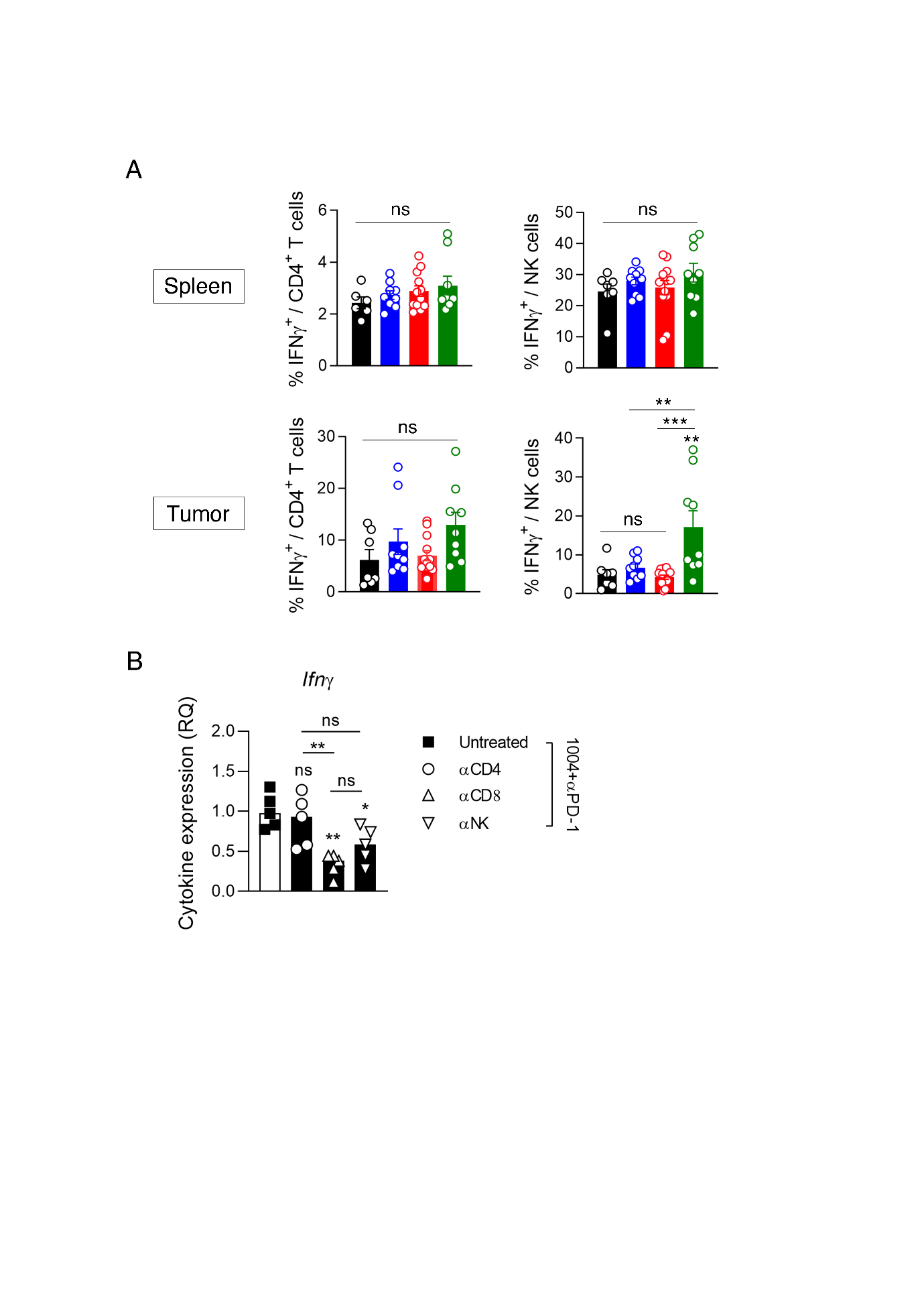
**

**Figure S7. IFNγ cytokine expression through combination therapy is dependent on CD8+ T cells among immune cells including CD4^+^ T cells, CD8^+^ T cells, and NK cells.**

**(A)** Lymphocytes isolated from the spleen and tumor of each group of mice at 7 days post treatment were re-stimulated in vitro. Representative plots are shown for expression of IFNγ on CD4^+^ T cells and NK cells. Data are pooled from three independent experiments (Control, n = 7; αPD-1, n = 9; CU06-1004, n = 13; Combination, n = 9). Statistical analysis by one-way ANOVA with Tukey’s multiple comparisons.

**(B)** qRT-PCR analysis of the inflammatory cytokine Ifnγ was performed after intravenous administration of αCD4, αCD8 or αNK1.1-depleted mAb to each group of mice before tumor injection (0.2 mg) and before treatment (0.2 mg). *n* = 5 per group. Statistical analysis by one-way ANOVA with Tukey’s multiple comparisons.

**
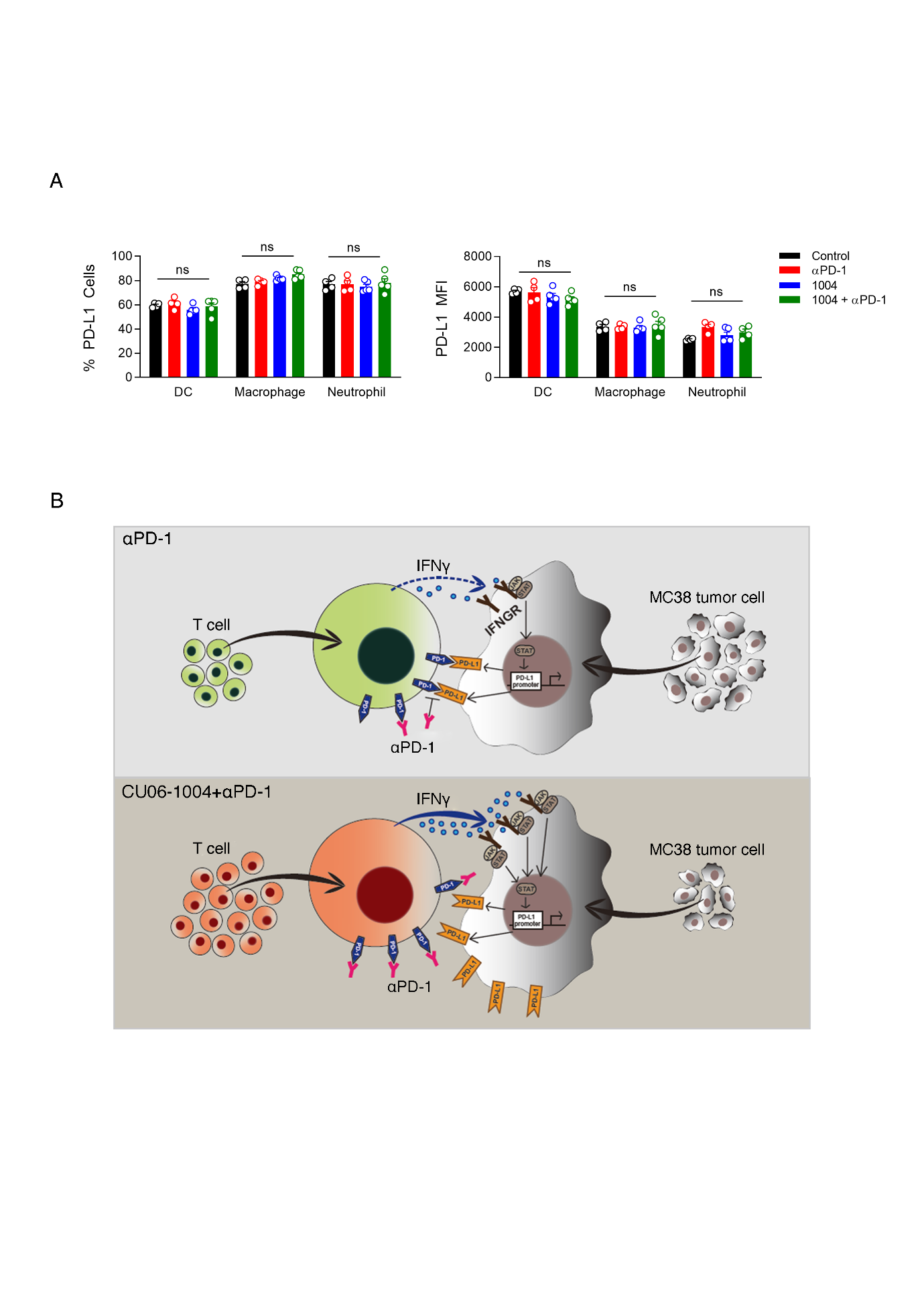
**

**Figure S8. Differences in PD-L1 between four groups were not seen in several innate lymphoid cells expressing PD-L1.**

**(A)** Flow cytometry analysis for PD-L1 staining of innate lymphoid cell in tumor-infiltrating lymphocytes. Quantification of the vessel density in the MC38 tumor center region and periphery region. *n* = 4-5 per group. Statistical analysis by one-way ANOVA with Tukey’s multiple comparisons.

**p* < 0.05; ***p* < 0.01; ****p* < 0.001. ns, not significant. Data represent ± SEM.

**(B)** In the presence of CU06-1004, infiltration of T cells and delivery of anti-PD-1 are enhanced to secrete a lot of cytokine such as IFNγ. IFNγ by T cells in the tumor binds to the IFNγ receptor within the tumor and upregulates the expression of PD-L1 by regulating STAT1.

**Figure SV1. Time-lapse movie showed direct changes in permeability by CU06-1004 in the Lewis lung cancer (LLC)-GFP-bearing BALB/c-nu/nu mice.**

Time lapse movie produced a dorsal skinfold chamber model to show the change in permeability when comparing in real time groups with and without CU06-1004. It was seemed that tumor vascular permeability was significantly reduced in the group treated with CU06-1004 compared with the vehicle group not treated with CU06-1004 in the LLC model. The time-lapse movie was produced on the 4day after the first treatment of the vehicle or CU06-1004 drug. Green, FITC staining; Red, CD31 staining; blue, DAPI staining.
